# The Influence of National Antibiotic Consumption on *Neisseria Gonorrhoeae* Antibiotic Resistance in Norway, 2003–2024

**DOI:** 10.1093/infdis/jiag076

**Published:** 2026-02-11

**Authors:** Patricia Campbell

**Affiliations:** Department of Microbiology and Infection Control, Akershus University Hospital, Lørenskog, Norway; Institute of Clinical Medicine, University of Oslo, Oslo, Norway

## Abstract

**Objectives:**

To investigate whether national population-level antibiotic consumption influences antimicrobial resistance (AMR) in Norwegian *Neisseria gonorrhoeae* isolates. To explore metrics suitable for ecological AMR studies.

**Methods:**

Longitudinal Norwegian gonococcal susceptibility data (2003–2024) were analysed alongside national antibiotic consumption. Temporal trends were examined graphically and associations assessed using one-tailed Spearman rank correlations. Novel metrics—The “Susceptible Isolate Pressure Indicator” (SIPI) and “Wild-Type Isolate Pressure Indicator” (WIPI) ratios were introduced to characterize shifts in minimum inhibitory concentration (MIC) distributions within susceptible or wild-type ranges.

**Results:**

Strong positive, significant correlations were observed between consumption of the most widely used antibiotic classes in Norway—betalactamase-sensitive penicillins and tetracyclines—and gonococcal geometric mean MIC for benzylpenicillin (*ρ* = 0.776, *P* < .001) and tetracycline (*ρ* = 0.841, *P* < .001). Penicillin-class consumption was also significantly associated with betalactamase plasmid carriage (*ρ* = 0.637, *P* = .013), consistent with horizontal gene transfer from commensal flora.

**Conclusions:**

Even in a low-consumption European context, Norwegian antibiotic use appears to shape gonococcal resistance, possibly partly via gene uptake from commensal *Neisseria*. The SIPI and WIPI ratios describe susceptible-range MIC histogram shapes, and offer utility for AMR surveillance by capturing isolate flux.

## Teaser

Norwegian national antibiotic consumption, though low in Europe, correlates with gonococcal resistance to high-volume classes (penicillins, tetracyclines), possibly via genes from Neisseria commensals, under bystander selection. A novel AMR metric (SIPI ratio) aids understanding of selective pressure.

*Neisseria gonorrhoeae* has developed resistance to every antibiotic used in its treatment since the antibiotic era began [1]. Future antibiotics are likely to fail in similar time-frames, so understanding gonococcal antimicrobial resistance (AMR) epidemiology is critical to prevent gonorrhea from becoming untreatable.

**Figure 1.**
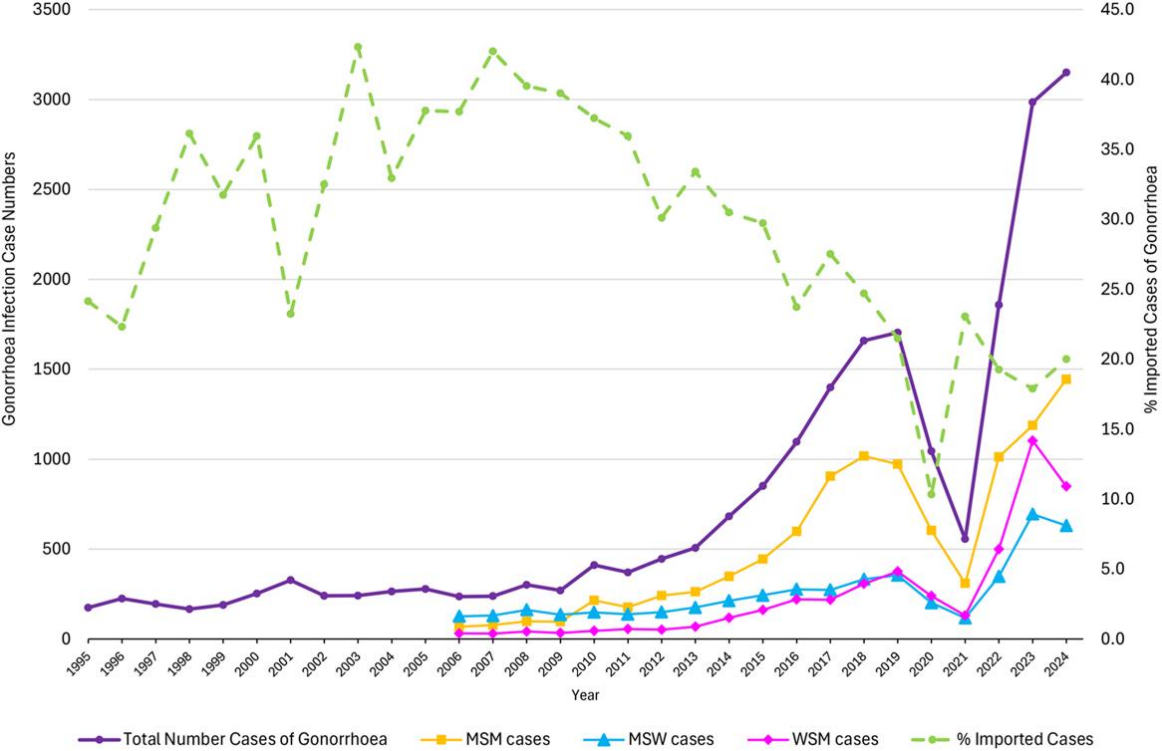
Annual total numbers of gonorrhea cases, by sexual orientation, with percentage of cases that were acquired outside of Norway. MSM = Men who have sex with men. MSW = Men who have sex with women. WSM = Women who have sex with men (data source [2]).

In Norway, post-COVID gonorrhea incidence is rising rapidly (Figure 1), but the proportion of cases acquired abroad has declined over two decades. There may then be a significant opportunity for national antibiotic stewardship to manage future development and persistence of *N. gonorrhoeae* AMR.

Many genetic determinants of gonococcal AMR originate in commensal pharyngeal *Neisseria* species, which are carried by all adult humans [3]. Universal carriage means that commensal Neisseria are exposed to every antibiotic that every citizen consumes. Population-level antibiotic consumption may therefore exert selective pressure on a society's commensal resistome, facilitating horizontal gene transfer (HGT) via transformation or conjugation during *N. gonorrhoeae* infection [3, 4].

In both European and global ecological analyses, Kenyon and colleagues have found correlations between population-level antibiotic consumption and *N. gonorrhoeae* AMR [5, 6]. A striking aspect of the European study is Norway's poor performance [5]. Despite relatively low antibiotic consumption, Norway had the highest European Geometric Mean Minimum Inhibitory Concentration (GMMIC) for azithromycin and ranked 4th and 5th highest for ceftriaxone and ciprofloxacin GMMICs, respectively [5]. This deserves closer investigation. In an editorial comment on the study, Olesen and Grad commended their innovative methods to elucidate whether direct or bystander selection mechanisms are involved [7]. Kenyon et al introduced a “right MIC shift indicator” (Susceptible isolate GMMIC correlated with the proportion of resistant isolates—“SGMMIC v. % resistance”), as a metric to detect whole MIC distribution shifts. This, they suggest, indicates *Neisseria* commensal “bystander” resistance gene transformation by *N. gonorrhoeae* [5].

In response to the surprising results for Norway, this retrospective, observational, experimental study uses publicly available, national data to re-examine the relationship between Norwegian antibiotic class consumption and AMR in *N. gonorrhoeae.* To answer Olesen and Grad's call for attention to AMR metrics for population-level studies, a novel “Susceptible Isolate Pressure Indicator” (SIPI) ratio is introduced.

## METHODS

### Data Sources

National gonorrhea, chlamydia and syphilis case numbers were obtained from the Norwegian Public Health Institute (NIPH) reporting system (MSIS) [2]. Antibiotic consumption data (Defined Daily Doses/1000 inhabitants/day, annualized by multiplying by 365) came from the Norwegian Surveillance for Antimicrobial Drug Resistance (NORM) drug wholesales reports [8]. Cephalosporin data included monobactams and carbapenems; macrolide data included lincosamides and streptogramins. Spectinomycin is not available in Norway, and figures are not available for the aminocyclitol class.

Thirteen years of susceptibility data were available. Twenty-four individual laboratories reported in 2003 and 2010, while national data were analyzed and reported in 2013–2015 by Oslo University Hospital and by the reference laboratory at NIPH from 2016 to 2024. Only single hospital data was available in 2018, so 2018 was excluded [8].

### MIC Definitions of Susceptibility

Resistance breakpoints followed Kenyon et al to allow comparison: ciprofloxacin (>0.064m mg/L), azithromycin (>0.5 mg/L), ceftriaxone/cefixime (>0.125 mg/L) [5]. Other antibiotics examined were spectinomycin (>64 mg/L), benzylpenicillin (>1 mg/L), and tetracycline (>1.0 mg/L, retained despite the 2022 European Committee on Antimicrobial Susceptibility Testing (EUCAST) change to >0.5 mg/L [9]). “Increased exposure” categorized isolates were grouped as susceptible.

Where available, EUCAST epidemiological cutoffs (ECOFFs) were used to define wild-type (WT) populations: ciprofloxacin 0.016 mg/L, azithromycin 1 mg/L, and spectinomycin 32 mg/L [9]. For ecological AMR studies to be fully comparable across time and geography, it is the more biologically correct wild-type (WT) that should be used. The WT distribution represents the inherent MIC variability seen in organisms with no phenotypically detectable resistance genes [10].

### Geometric Mean MIC Calculation

Weighted GMMIC and subgroup (susceptible, WT) GMMICs were calculated by the formula:


$$\mathrm{Weighted}\;\mathrm{GMMIC}=2^{\wedge }\left(\sum (wi\times \mathrm{lo}g_{2}(xi))/\sum wi\right)$$

where *xi* = MIC (mg/L), *wi* = proportion of isolates (*p*/100). Subgroups retained original percentages for each MIC value in the susceptible or WT ranges. Data was truncated at 0.004 mg/L and 128 mg/L for cefixime, tetracycline, spectinomycin and azithromycin, and 0.004 mg/L and 32 mg/L for benzylpenicillin, ceftriaxone and ciprofloxacin.

### SIPI and WIPI Ratios

The SIPI ratio is the quotient of SGMMIC divided by the susceptible isolate mode. The WIPI analogously uses WT values. It represents a mathematical means of describing the appearance of the MIC distribution histogram in the susceptible zone. Where the mode is equal to the SGMMIC, as would be seen in a purely symmetrical MIC histogram, the ratio = 1. Where the ratio is > 1, there is a significant subpopulation of isolates with higher MICs breaking away from the most frequent (modal) MIC value, heading for resistance. Where the ratio is < 1, the mode is greater than the mean MIC. This may represent a sudden proliferation of “super-fit” clones with higher MICs that shift the susceptible zone modal MIC. It may also represent a laboratory technical problem. An assessment of the validity of Norwegian data is therefore included. Norwegian WT or susceptible isolate modes were plotted with EUCAST equivalents, applying Kahlmeter and Turnidge's EUCAST principle that “the wild-type mode does not change over time” [10]. The EUCAST WT or susceptible modes were defined from equally weighted aggregated distributions [9] using the higher MIC when modes were equal.

### Analysis

**Figure 2.**
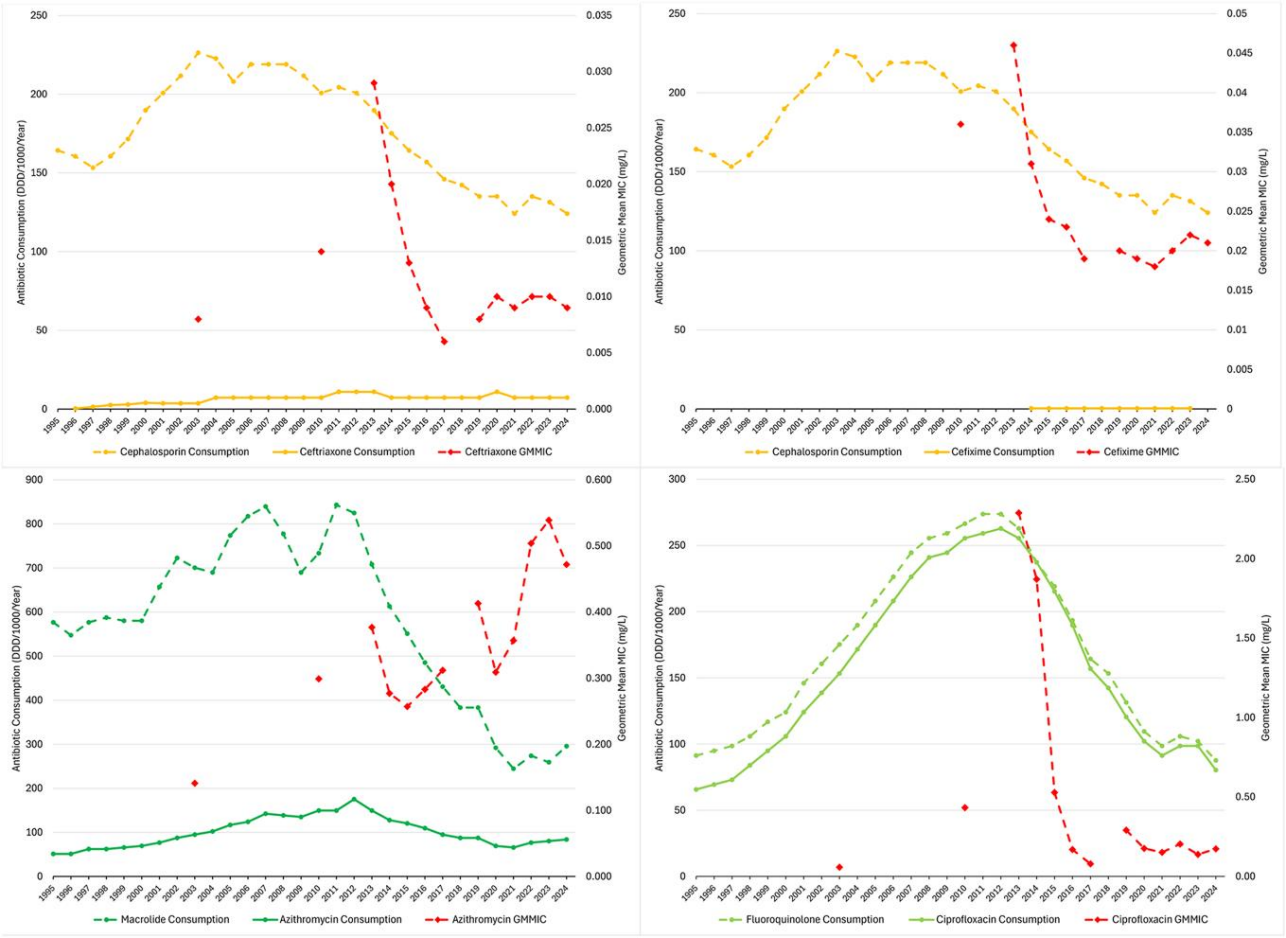
Annual antimicrobial consumption (Daily Defined Doses/1000 inhabitants/Year) of gonorrhea treatment antibiotics and their class v. *N. gonorrhoeae* treatment antibiotic Geometric Mean Minimum Inhibitory Concentrations (mg/L).

Graphs of GMMIC v. antibiotic consumption trends (Figure 2, Supplementary Figure 1) were compared with Kenyon et al.'s corresponding supplementary plots [5].

**Figure 3.**
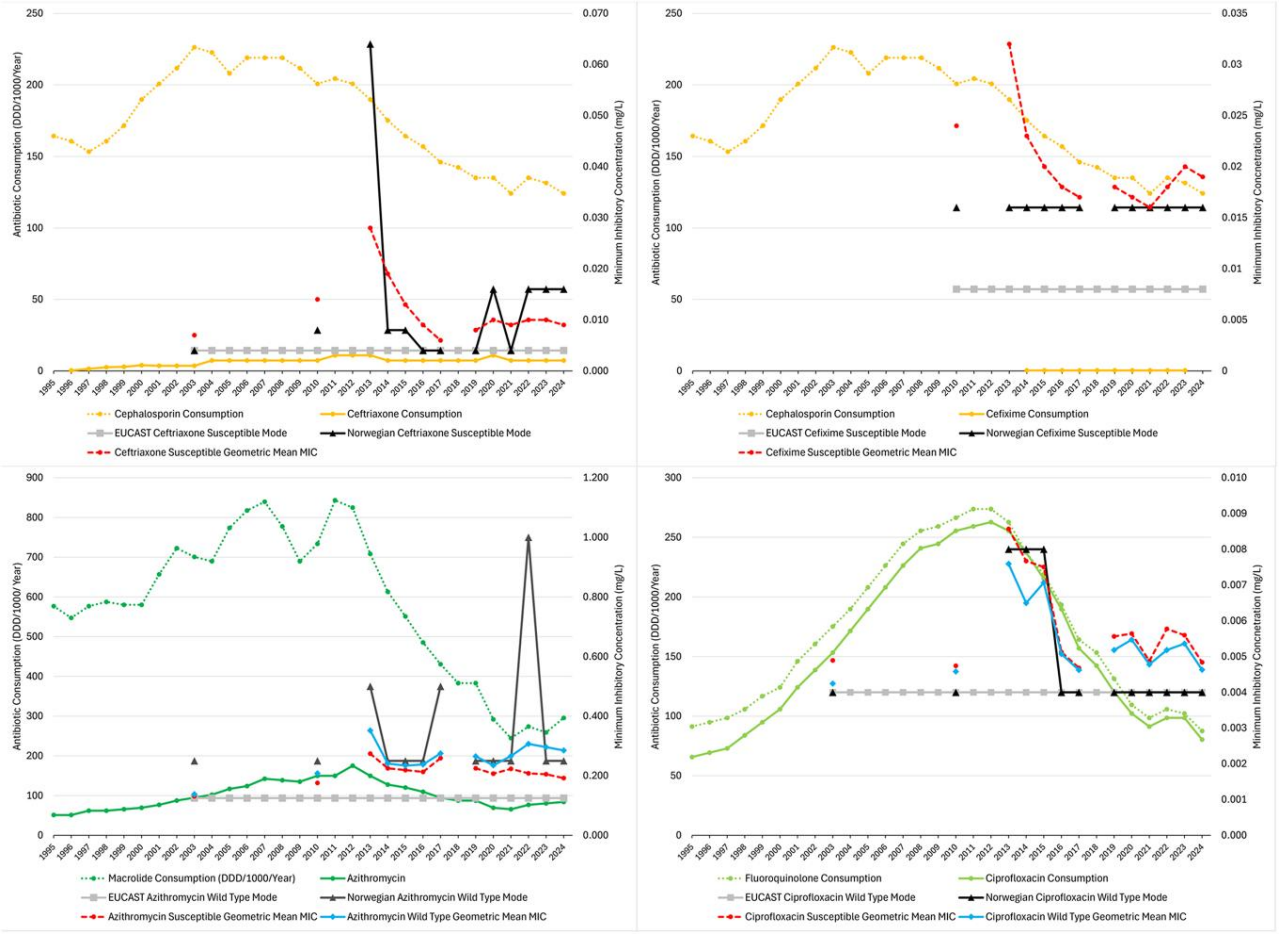
Annual antimicrobial consumption (Daily Defined Doses/1000 inhabitants/Year) v. *N. gonorrhoeae* Susceptible and Wild-Type Geometric Mean Minimum Inhibitory Concentrations (mg/L), with the European Committee on Antimicrobial Susceptibility Testing mode and Norwegian Modal Minimum Inhibitory Concentrations (mg/L).

For quality control, SGMMIC and WTGMMICs (where ECOFFS have been defined) were plotted with susceptible and WT modes (EUCAST and Norwegian, Figures 3 and S2). The modes served as controls as they should remain stable, and the Norwegian mode should not deviate from EUCAST by more than one MIC dilution step [9].

**Figure 4.**
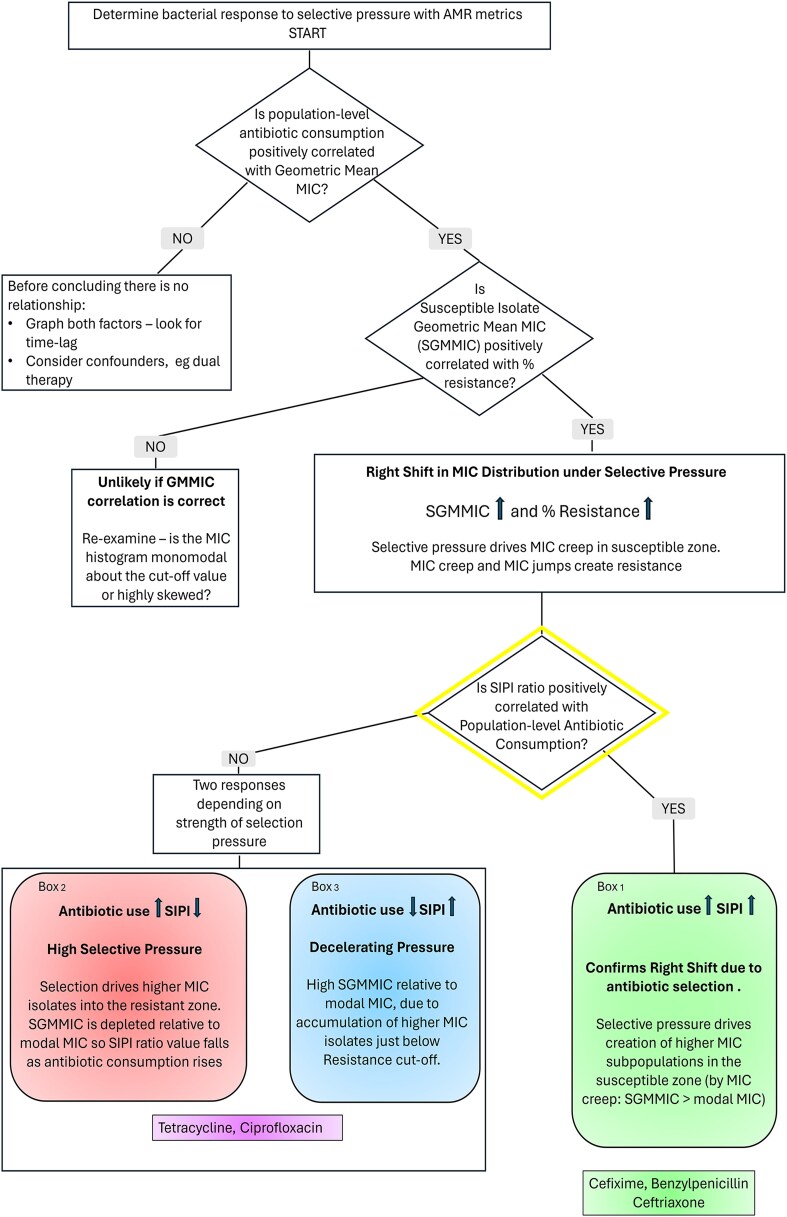
Sorting of gonococcal isolate movement in response to antibiotic selective pressure. Data from Tables 1–3.

One-tailed Spearman rank correlations assessed antibiotic class consumption versus treatment antibiotic GMMIC, SGMMIC, % AMR, and SIPI ratio (Table 1). The data was not time-lagged. Bonferroni correction was applied (*P* < .0021). Further, betalactamase status was correlated separately with penicillin consumption by one-tailed Spearman analysis. SGMMIC versus % AMR, WTGMMIC versus % non-WT, (Table 2), and SIPI and WIPI ratio versus % AMR were also correlated (Table 3) to detect MIC shifts. Figure 4 provides a summary flowchart, to facilitate visualization of the gonococcal response to selection pressure.

**Table 1.** One-tailed Spearman Correlation Analysis of the Relationship Between Antibiotic Class Consumption (Daily Defined Doses/1000 Inhabitants/Year) and Resistance Metrics for the Homologous Antibiotic on the Standard Laboratory Test-panel

		Geometric Mean MIC		Geometric Mean MIC For Susceptible Isolates		% Antimicrobial Resistance		Susceptible Isolate Pressure Indicator (SIPI)	
Antibiotic	Antibiotic Class Consumption	Correlation Coefficient (95% CI)	*P*	Correlation Coefficient (95% CI)	*P*	Correlation Coefficient (95% CI)	*P*	Correlation Coefficient (95% CI)	*P*
Ceftriaxone	Cephalosporins	.331 (−.190, 1.000)	.135	.308 (−.214, 1.000)	.153	.515 (.034, 1.000)	.036	.199 (−.322, 1.000)	.257
Cefixime	Cephalosporins	.768 (.423, 1.000)	.002	.649 (.206, 1.000)	.011	.701 (.295, 1.000)	.006	.649 (.206, 1.000)	.011
Azithromycin	Macrolides	−.632 (−1.000, −.206)	.010	.033 (−.464, 1.000)	.457	−.621 (−1.000, −.189)	.012	−.319 (−1.000, .202)	.144
Ciprofloxacin	Fluoroquinolones	.560 (.098, 1.000)	.023	.275 (−.248, 1.000)	.182	.454 (−.046, 1.000)	.060	−.582 (−1.000, −.130)	.018
Tetracycline	Tetracyclines	.841 (.597, 1.000)	**<.001**	.612 (.174, 1.000)	.013	.787 (.483, 1.000)	**<**.**001**	−.360 (−1.000, .157)	.113
Benzylpenicillin	Betalactamase- sensitive penicillins	.776 (.461, 1.000)	**<**.**001**	.253 (−.270, 1.000)	.202	.715 (.347, 1.000)	.003	.363 (−.154, 1.000)	.111

Bold = *P* significant at the 0.0021 level (Bonferroni adjustment), *n* = 13 years for all antibiotics except cefixime *n* = 12 Years.

**Table 2.** One-tailed Spearman Rank Correlation Analysis Between the Susceptible Isolate Geometric Mean Minimum Inhibitory Concentration (SGMMIC) or Wild-type Geometric Mean Minimum Inhibitory Concentration (WTGMIC), and the Percentage of Resistant or Non-wild-type Isolates

	SGMMIC v % Resistance		WTGMMIC v % Non-WT	
Antibiotic	Correlation Coefficient (95% CI)	*P*	Correlation Coefficient (95% CI)	*P*
Ceftriaxone	.352 (−.167, 1.000)	.119		
Cefixime	.468 (−.057, 1.000)	.062		
Azithromycin	−.080 (−1.000, .426)	.398	.479 (−.014, 1.000)	.049
Ciprofloxacin	.638 (.216, 1.000)	.009	.626 (.197, 1.000)	.011
Tetracycline	.663 (0,257, 1.000)	.007		
Benzylpenicillin	.258 (−.265, 1.000)	.197		

Bonferroni-adjusted significance at *P* < .00625, *n* = 13 years for all antibiotics except cefixime *n* = 12 years.

**Table 3.** Susceptible or Wild-Type Pressure Indicator (SGMMIC/Susceptible Mode, or WTGMMIC/WT Mode) v. % AMR

	Susceptible Isolate Pressure Indicator v. % Resistance		Wild-type Isolate Pressure Indicator v. % Non-WT	
Antibiotic	Correlation Coefficient (95% CI)	*P*	Correlation Coefficient (95% CI)	*P*
Ceftriaxone	−.086 (−1.000, .421)	.390		
Cefixime	.468 (−.057, 1.000)	.062		
Azithromycin	.102 (−.408, 1.000)	.37	.418 (−.090, 1.000)	.078
Ciprofloxacin	−.435 (−1.000, .070)	.069	−.445 (−1.000, .057)	.064
Tetracycline	−.532 (−1.000, −.057)	.031		
Benzylpenicillin	.357 (−.161, 1.000)	.115		

Bonferroni-adjusted significance at *P* < .00625), *n* = 13 years for all antibiotics except cefixime *n* = 12 years.

Gonorrhea, chlamydia, and syphilis case numbers were correlated using two-tailed Spearman rank correlation, to elucidate antibiotic treatment burdens.

Data handling and analysis were performed in Statistical Package for the Social Sciences (SPSS) IBM SPSS Statistics for Windows, version 26 (IBM Corp., Armonk, N.Y., USA) and Microsoft Excel version 2408. Grok 4 artificial intelligence was taught to do the GMMIC calculations, results were double-checked by the author and an independent mathematician.

## RESULTS

Figure 2 plots annual antimicrobial consumption (Daily Defined Doses (DDD)/1000 inhabitants/year) versus *N. gonorrhoeae* GMMICs (mg/L) for the four antimicrobials reported by Kenyon et al. The remaining antibiotics are presented in Supplementary Figure 1. Doxycycline consumption is included with tetracyclines as it is used for Chlamydia trachomatis treatment and is increasingly used as doxycycline post-exposure prophylaxis (‘doxy-pep’).

Antibiotic consumption has decreased for all antibiotics since 2012. GMMIC trends for the cephalosporins, ciprofloxacin, tetracycline and benzylpenicillin showed concordant pre- and post-COVID trends. Azithromycin GMMIC exhibited a rising saw-tooth pattern, resembling the trend in earlier macrolide consumption.

Overall mean GMMICs (2003–2024, mg/L) were: ceftriaxone: 0.012; cefixime: 0.025; ciprofloxacin: 0.505; azithromycin: 0.349; tetracycline: 1.121; benzylpenicillin: 0.730; spectinomycin: 13.546.

Figure 3 and Supplementary Figure 2 highlight Norwegian susceptible and WT mode variance from EUCAST—two dilution steps for tetracycline and benzylpenicillin, and three for ceftriaxone and azithromycin. There were apparent laboratory-dependent shifts (ciprofloxacin 2013–2015, Oslo University Hospital). The Norwegian mode of cefixime is consistently one step above the EUCAST susceptible mode. Norwegian spectinomycin mode variance from EUCAST in 2010 clearly influences S/WTGMMIC (Supplementary Figure 2).

Strong positive correlations were observed between consumption of antibiotic class and homologous antibiotic GMMIC for betalactamase-sensitive penicillins benzylpenicillin (*ρ* = 0.776, *P* < .001), and tetracyclines-tetracycline (*ρ* = 0.841, *P* < .001). Cephalosporins-cefixime just missed statistical significance (*ρ* = 0.768, *P* = .002).

Wild-type v. antibiotic consumption data, not shown in the table due to lack of space:

Macrolides v. Azithromycin WTGMMIC: *ρ* = −0.440, *P* = .066, (CI: −1.000, .064). % non-WT: −0.657, *P* = .007, (CI: −1.000, −.248). WIPI: *ρ* = −0.445, *P* = .064, (CI: −1.000, .057).

Fluoroquinolones v. Ciprofloxacin WTGMMIC: *ρ* = 0.187, *P* = .271, (CI:−.333, 1.000). % non-WT *ρ* = 0.456, *P* = .059, (CI: −.043, 1.000), WIPI *ρ* = −0.670, *P* = .006, (CI: −1.000, −.269).

Penicillin-class consumption was significantly associated with betalactamase plasmid carriage (at *P* < .05): *ρ* = 0.637, *P* = .013 (CI .187, 1.000).

Table 2 shows positive “right shift” metric correlations for all antibiotics except azithromycin. However, for azithromycin the analogous wild-type metric “WTGMMIC versus % non-WT” correlation is positive (*ρ* = 0.479, *P* = .049) and similar to the shift seen in cefixime (*ρ* = 0.468). No tests reached statistical significance.

Table 3 revealed positive correlations between SIPI or WIPI v. % AMR for azithromycin, cefixime and benzylpenicillin, and moderately strong negative correlations with ciprofloxacin, tetracycline. No tests achieved statistical significance.

Figure 4 brings together the gonococcal correlation coefficient responses to antibiotic selection pressure, by asking three main questions (in diamond nodes). By applying the SIPI ratio correlation with antibiotic consumption, (yellow diamond) we can go further than Kenyon et al's metrics in determining isolate shift. Azithromycin falls out at the first question, so it is not placed on the flowchart.

Lastly, annual case numbers of gonorrhea were significantly and strongly positively correlated with cases of chlamydia (*n* = 19 years, two-tailed Spearman *ρ* = 0.891, *P* < .001, CI .727–.959) and syphilis (*n* = 29 years, two-tailed *ρ* = 0.887, *P* < .001, CI .766 −.947).

## DISCUSSION

This study failed to replicate the findings of Kenyon et al for Norwegian azithromycin resistance. Norway was presented as a European outlier GMMMIC of 0.63 mg/L in 2010–2016 [5]. National data for 2011 or 2012 were not available to us, but our mean GMMIC of 0.299 mg/L (2010–2016) and overall mean 0.349 mg/L (2003–2024) would place Norway with other Northern European countries with low azithromycin consumption.

Some variation in results is to be expected, given that our data is the comprehensive national annual dataset, while Kenyon et al's study is based upon European Gonococcal Antimicrobial Surveillance Programme (Euro-GASP) sentinel data, collected each year in April/May and October/November [11]. However, Cole et al found high concordance between Norway's national azithromycin susceptibility data and Euro-GASP's in 2009–2013, (though Norway only contributed 2 years of data to that study) [12]. Further, concordance between Norwegian Euro-GASP and national azithromycin resistance data was reasonable for 2015 (3.6% vs 6.6%), 2016 (16.2% vs 11.5%), and 2019 (16.0% vs 19.4% at the 1 mg/L cutoff) [8, 13–15]. Norway takes part in the Euro-GASP External Quality Assessment (EQA) scheme and performs adequately [16]. This study therefore questions Norway's top position in Kenyon et al's azithromycin resistance ranking, and the outlier position may have impacted their regression analyses.

### Is Norwegian Population-Level Antibiotic Consumption Reflected in Gonococcal AMR?

Kenyon et al found significant associations between European population-level antibiotic use and gonococcal resistance to cephalosporins and quinolones, but not azithromycin [5].

This study found significant, positively correlated GMMICs with population-level consumption of the homologous antibiotic class for benzylpenicillin and tetracycline (*ρ* = 0.776, and *ρ* = 0.841, respectively, *P* < .001). Despite strong positive correlations for cephalosporins-cefixime (*ρ* = 0.768, *P* = .002) and fluoroquinolones-ciprofloxacin (*ρ* = 0.560, *P* = .023), these did not reach statistical significance at strict Bonferroni corrected significance levels. Weak results for ceftriaxone GMMIC may be partly due to confounding with azithromycin, as was the intention through the practice of dual treatment, from c. 2010–2020.

### Is the SIPI Ratio a Useful Metric?

Olesen and Grad called for the development of “informative and comparable surveillance metrics” for ecological AMR studies, and argued that, in underpowered population-level studies, correlation strength may be more informative than statistical significance alone [7].

The SIPI and WIPI ratios improve upon traditional AMR surveillance metrics. By understanding the isolate flux driving positive and negative correlation coefficients, we can map and quantify “MIC creep” histogram shape-shifts under selective pressure.

Figure 4 provides a framework for how SIPI and WIPI ratios can be used. If we start with GMMIC (the summary measure of the central tendency of MIC values), we see that in our dataset only Azithromycin does not have a positive correlation with antibiotic consumption. Further, all remaining antibiotics show positive Kenyon right shift “SGMMIC v. % resistance” correlations.

This is useful information, but we gain more insight at this point by applying question 3 (yellow diamond): “Is SIPI positively correlated with antibiotic consumption?”. This produces two “camps’ of isolate response to selective pressure.

### Positive “SIPI v. Antibiotic Consumption” Correlation: Expansion in the Susceptible Zone

Positive “SIPI ratio v. antibiotic consumption” correlations in cefixime (*ρ* = 0.649), ceftriaxone (*ρ* = 0.199), and benzylpenicillin (*ρ* = 0.363) indicate that antibiotic consumption drives creation of higher-MIC subpopulations in the susceptible zone, in a monotonic relationship, as described in Green Box 1 (Figure 4). Interestingly, all the betalactam antibiotics fall together, despite major differences in population-level consumption of these classes [8]. Betalactamase-sensitive penicillins are historically the class consumed in the greatest volumes in Norway, in human medicine, but also in food animals and companion pets [8] (which may also impact on the human resistome) [17]. Cephalosporin consumption is < 2% of the national total human antibiotic usage. The betalactam cluster response may represent shared fitness costs of mutations affecting both antibiotic classes (changes in Penicillin Binding Protein 2 (*penA*), efflux mechanisms (*mtrR*) and porins (*penB*) [1]). It may also indicate the degree of ease of transfer of these genes from commensal *Neisseria*. Another explanation beyond new uptake of genes by HGT is the selective expression of genes already accumulated. Decades of contact between the gonococcus, *Neisseria* commensals and penicillins may have saturated penicillin resistance gene accumulation in lineages of gonococci, limiting further gains to preserve fitness. This is supported by benzylpenicillin having the lowest “SGMMIC v % resistance” right shift (*ρ* = 0.258) despite having the second highest GMMIC v. antibiotic consumption (*ρ* = 0.776). The dynamic is one of high resistance, but relatively little rightward MIC creep. The gonococci benefit further from the co-resistance to cephalosporins conferred by these genes.

### Negative SIPI v. Antibiotic Consumption Correlation: Depletion of the Susceptible-Range

Negative “SIPI ratio v. antibiotic consumption” correlations (ciprofloxacin *ρ* = −0.582, and tetracycline *ρ* = −0.360), suggest dynamic gonococcal responses to changing selection pressure (boxes 2 and 3, Figure 4). At high sustained selective pressure (red box 2) the susceptible higher MIC subpopulation shifts rightwards to the degree that higher MIC isolates are lost to the resistant zone, leaving the susceptible population depleted. The SGMMIC falls closer to the modal MIC and the SIPI ratio falls. If pressure eases after prolonged high exposure (blue box 3), the selection pressure that previously pushed these higher MIC variants into the resistant zone weakens, allowing high MIC susceptible isolates to persist and accumulate in the susceptible zone. The SGMMIC rises relative to the mode, so the SIPI ratio rises as antibiotic selection pressure falls.

### Linking Susceptible-Range Shifts to Resistance Emergence

Under selective pressure, isolates will either become more resilient or disappear due to lack of fitness. We can check for coherence in the fate of isolates from Figure 4, by examining the “SIPI v % Resistance correlation”:

Ciprofloxacin (*ρ* = −0.435), and tetracycline (*ρ* = −0.532) maintain negative correlations, indicating loss of high MIC susceptible isolates to the resistant zone: SIPI falls as % resistance rises, as predicted above.

Cefixime (*ρ* = 0.468), Benzylpenicillin (*ρ* = 0.357); there is concurrent expansion of higher-MIC susceptibles, and rising resistance. Ceftriaxone is unclassifiable, probably due to persistently low resistance levels.

### The Case of Azithromycin

As Figure 2 reveals, macrolide consumption swings periodically—this is due to Europe-wide epidemics of Mycoplasma pneumoniae, every 4−7 years [8, 18]. Our uniquely strange negative GMMIC and % AMR correlation coefficients for azithromycin are due to the inadequacy of trying to fit a monotonic relationship to an unlagged, fluctuating data trend. Kenyon et al did not take account of this Europe-wide periodicity either. Further, it is essential that the wild-type (ECOFF 1 mg/L) be used for surveillance studies of azithromycin. The “susceptible” range based on the previous “clinical breakpoint” of 0.5 mg/L arbitrarily split the wild-type distribution [9]. When we examine Azithromycin's wild-type “MIC creep” metrics, (which are internally consistent with each other, and not affected by lack of lagging with antibiotic consumption), we see coefficients of *ρ* = 0.479 (WTGMMIC v % non-WT), and *ρ* = 0.418 (WIPI v % non-WT), respectively. This suggests a gonococcal response to macrolide selective pressure in the same range as to cefixime.

### Can We Determine Dominant Adaptive Mechanisms?

Kenyon et al propose that “right MIC shift” is due to gene transfer from resistant *Neisseria* commensals. However, whole MIC distribution right shift is only a measure of MIC creep. This may also be due to cumulative direct mutations, or gonococcal adaptive resistance to anatomical niche pH, oxygen levels [19, 20] and sex hormones in the genital tract [21]. The significant positive correlation between penicillin-class consumption and betalactamase-positive isolates may be the best indicator we have of commensal-derived plasmid conjugation with N. gonorrhoeae (*ρ* = 0.637, *P* = .013).

Beyond population-level antibiotic consumption, treatments for other sexually transmitted infections may act to maintain penicillin and tetracycline resistance genes in the gonococcus. This is an example of the ecological fallacy, as patients with gonorrhea are possibly not representative of the general population in antibiotic exposure. There is a high burden of chlamydia infection in Norway [22], and doxycycline post-exposure prophylaxis (‘doxy-pep’) is another source of tetracycline exposure, particularly in the MSM group. Both syphilis (treated with the betalactamase-sensitive, long-acting benzathine-penicillin) and chlamydia case numbers are highly correlated with gonorrhea case numbers (*ρ* = 0.887, *P* < .001, and *ρ* = 0.891, *P* < .001, respectively). The high right-shift correlation (Table 2) for ciprofloxacin (*ρ* = 0.638, *P* = .009) may reflect fluoroquinolone (moxifloxacin) treatments for macrolide-resistant Mycoplasma genitalium infections (case numbers not recorded). The treatment burden of other STIs in this patient group is likely to represent an additional selective pressure upon their microbiomes, higher than that experienced by the general population.

This study lacked sufficient years of data to allow multivariate analysis of other factors impacting AMR. Import from countries with different antibiotic stewardship histories is likely to be an important source of resistance genes. When infected abroad, MSM are more commonly infected in Europe, while consistently high proportions of MSW continue to be infected in Thailand and the Philippines [22], areas of AMR concern [23]. Our data follows the same patterns for European susceptibility of ceftriaxone, cefixime and azithromycin [15]—suggesting that global sexual network-driven clonal expansion is important. We examined one antibiotic at a time, but the gonococcus is known to exhibit the ability to carry multiple resistance genes at once—therefore consumption of one class may “accidentally” select for a whole other class through antibiotic co-resistance [24]. Antibiotics’ varying half-lives, ecological shadows [25, 26], duration of use in front-line gonorrhea treatment and time since abandonment, as well as the impact of non-antibiotic drugs (eg, antidepressants) on the human microbiome may further confound results [27].

In addition, technical issues clearly affect the reporting of many antibiotics (Figure 3, Supplementary Figure 2). It is not known if the swings in Norwegian susceptible and WT modes from EUCAST modes affect the entire MIC range, but they are concerning and may undermine our results and Euro-GASP data, especially for antibiotics like ceftriaxone, where the greater weight of the MIC distribution lies in the susceptible zone. As the susceptible mode and the SGMMIC move together, however, the SIPI ratio is still valid.

In conclusion, Norwegian antibiotic stewardship should maintain efforts to reduce population-level antibiotic consumption for the sake of the societal resistome. Behavioral interventions to prevent import of multi-resistant gonorrhea infection will become ever more important with the coming launch of the new antibiotics zoliflodacin and gepotidacin [28]. The novel SIPI and WIPI ratios give insights to adaptive gonococcal dynamics that would be invisible with traditional surveillance metrics. They should be tested in future, larger, ecological AMR surveillance studies.

## Supplementary Material

jiag076_Supplementary_Data

## Supplementary Data

Supplementary materials are available at *The Journal of Infectious Diseases* online (http://jid.oxfordjournals.org/). Supplementary materials consist of data provided by the author that are published to benefit the reader. The posted materials are not copyedited. The contents of all supplementary data are the sole responsibility of the authors. Questions or messages regarding errors should be addressed to the author.
