# Supplementary material for: The Influence of National Antibiotic Consumption on Neisseria Gonorrhoeae Antibiotic Resistance in Norway, 2003–2024
Source: J Infect Dis. 2026 Feb 11;233(5):e1193–202. doi: 10.1093/infdis/jiag076 (PMC13175608; doi:10.1093/infdis/jiag076)
Supplement: jiag076_Supplementary_Data [file jiag076_supplementary_data.zip › CampbellSupplementFigure1TITLEandLEGEND.docx]

**Supplementary Figure 1. Antimicrobial Consumption and Geometric Mean MIC**

Figure S1. Annual antimicrobial consumption (Daily Defined Doses/1000 inhabitants/Year) of treatment antibiotics and their class v. *N. gonorrhoeae* Geometric Mean Minimum Inhibitory Concentrations (mg/L).
