# Supplementary material for: The Influence of National Antibiotic Consumption on Neisseria Gonorrhoeae Antibiotic Resistance in Norway, 2003–2024
Source: J Infect Dis. 2026 Feb 11;233(5):e1193–202. doi: 10.1093/infdis/jiag076 (PMC13175608; doi:10.1093/infdis/jiag076)
Supplement: jiag076_Supplementary_Data [file jiag076_supplementary_data.zip › CampbellSupplementFigure2TITLEandLEGEND.docx]

**Supplementary Figure 2. Quality control of Norwegian data by comparison of Norwegian and EUCAST modes**

Figure S2. Annual antimicrobial consumption (Daily Defined Doses/1000 inhabitants/Year) v. *N. gonorrhoeae* Susceptible and Wild Type Geometric Mean Minimum Inhibitory Concentrations (mg/L), with the European Committee on Antimicrobial Susceptibility Testing mode and Norwegian Modal Minimum Inhibitory Concentrations (mg/L). No aminocyclitol/spectinomycin consumption data.
