# Supplementary figures and images for: The Influence of National Antibiotic Consumption on Neisseria Gonorrhoeae Antibiotic Resistance in Norway, 2003–2024
Source: J Infect Dis. 2026 Feb 11;233(5):e1193–202. doi: 10.1093/infdis/jiag076 (PMC13175608; doi:10.1093/infdis/jiag076)

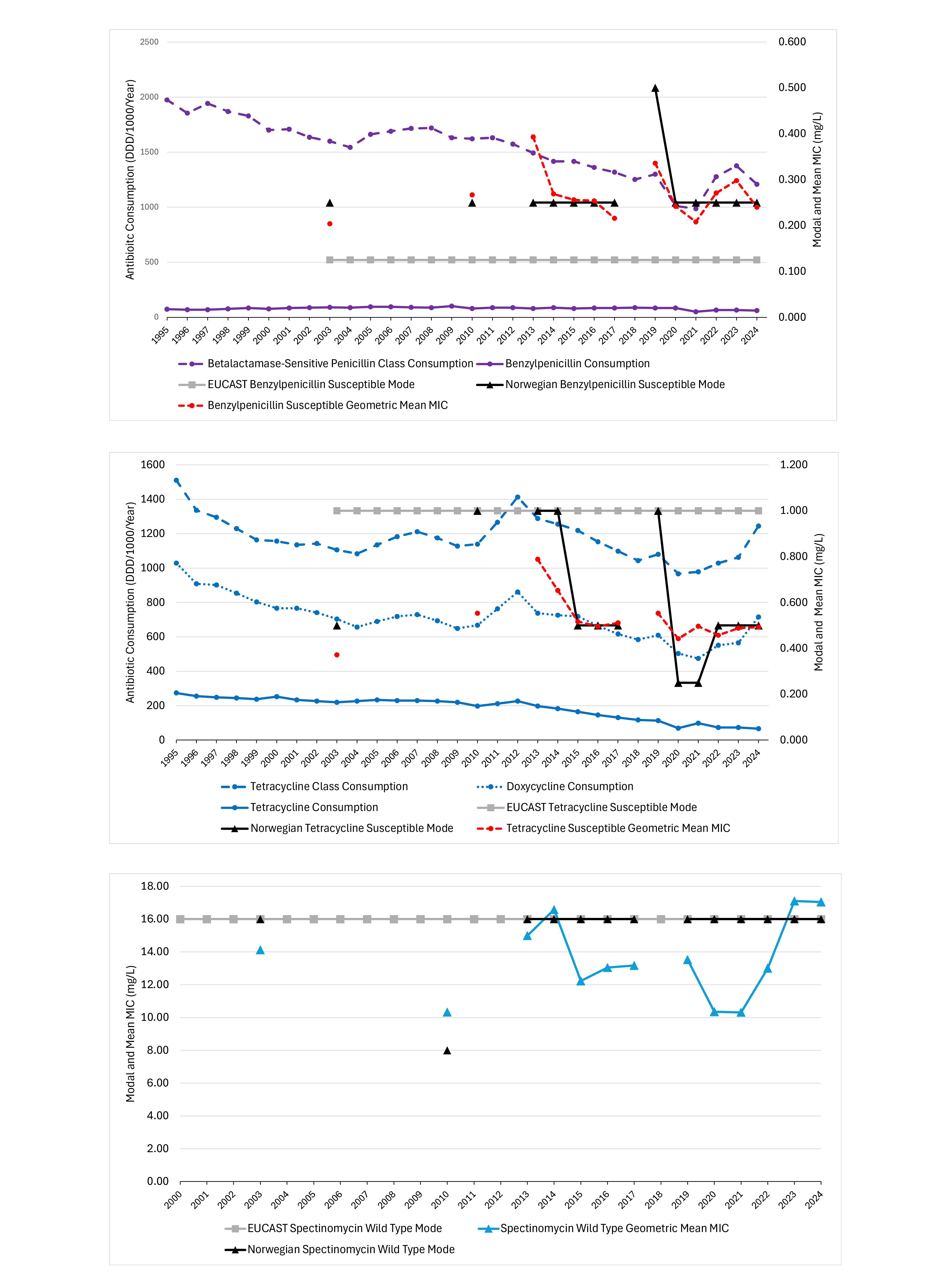

Supplement: jiag076_Supplementary_Data [file jiag076_supplementary_data.zip › CampbellSuppFigS2.tif]
